# Alveolus analysis: a web browser-based tool to analyze lung intravital microscopy

**DOI:** 10.1186/s12890-022-02274-7

**Published:** 2022-12-17

**Authors:** Alexander L. Politowicz, Andrew T. Burks, Yushen Dong, Yu Maw Htwe, Steven M. Dudek, G. Elisabeta Marai, Patrick Belvitch

**Affiliations:** 1 Department of Computer Science, College of Engineering, University of Illinois Chicago, Chicago, IL USA; 2 Department of Mathematics, Statistics, and Computer Science, University of Illinois Chicago, Chicago, IL USA; 3 Division of Pulmonary, Critical Care, Sleep and Allergy, Department of Medicine, University of Illinois Chicago, CSB 915, 840 S. Wood St., Chicago, IL 60612 USA

**Keywords:** Acute lung injury, Acute respiratory distress syndrome, ARDS, Inflammatory lung injury, Two-photon microscopy, Intravital lung microscopy, Image processing, Web browser-based interface, Temporal data visualization

## Abstract

**Background:**

Acute lung injury and the acute respiratory distress syndrome are characterized by pulmonary inflammation, reduced endothelial barrier integrity and filling of the alveolar space with protein rich edema fluid and infiltrating leukocytes. Animal models are critical to uncovering the pathologic mechanisms of this devastating syndrome. Intravital imaging of the intact lung via two-photon intravital microscopy has proven a valuable method to investigate lung injury in small rodent models through characterization of inflammatory cells and vascular changes in real time. However, respiratory motion complicates the analysis of these time series images and requires selective data extraction to stabilize the image. Consequently, analysis of individual alveoli may not provide a complete picture of the integrated mechanical, vascular and inflammatory processes occurring simultaneously in the intact lung. To address these challenges, we developed a web browser-based visualization application named Alveolus Analysis to process, analyze and graphically display intravital lung microscopy data.

**Results:**

The designed tool takes raw temporal image data as input, performs image preprocessing and feature extraction offline, and visualizes the extracted information in a web browser-based interface. The interface allows users to explore multiple experiments in three panels corresponding to different levels of detail: summary statistics of alveolar/neutrophil behavior, characterization of alveolar dynamics including lung edema and inflammatory cells at specific time points, and cross-experiment analysis. We performed a case study on the utility of the visualization with two members or our research team and they found the tool useful because of its ability to preprocess data consistently and visualize information in a digestible and informative format.

**Conclusions:**

Application of our software tool, Alveolus Analysis, to intravital lung microscopy data has the potential to enhance the information gained from these experiments and provide new insights into the pathologic mechanisms of inflammatory lung injury.

**Supplementary Information:**

The online version contains supplementary material available at 10.1186/s12890-022-02274-7.

## Background

The acute respiratory distress syndrome (ARDS) is characterized by acute inflammatory lung injury and is a common cause of respiratory failure with significant morbidity and mortality [1]. The pathophysiology of ARDS is characterized by a dysfunctional alveolar-capillary barrier and flooding of the normally air-filled alveolar space with protein rich edema fluid which impairs normal gas exchange [2, 3]. ARDS is caused by various infectious and inflammatory insults such as pneumonia or systemic infection with bacteria or viruses [4] including COVID-19 [5]. Animal models of lung injury are important in revealing the molecular and cellular mechanisms of disease progression [6]. In particular, experimental mouse models of ARDS have been employed successfully to study features such as lung edema formation and leukocyte infiltration [7].

**Fig. 1:**
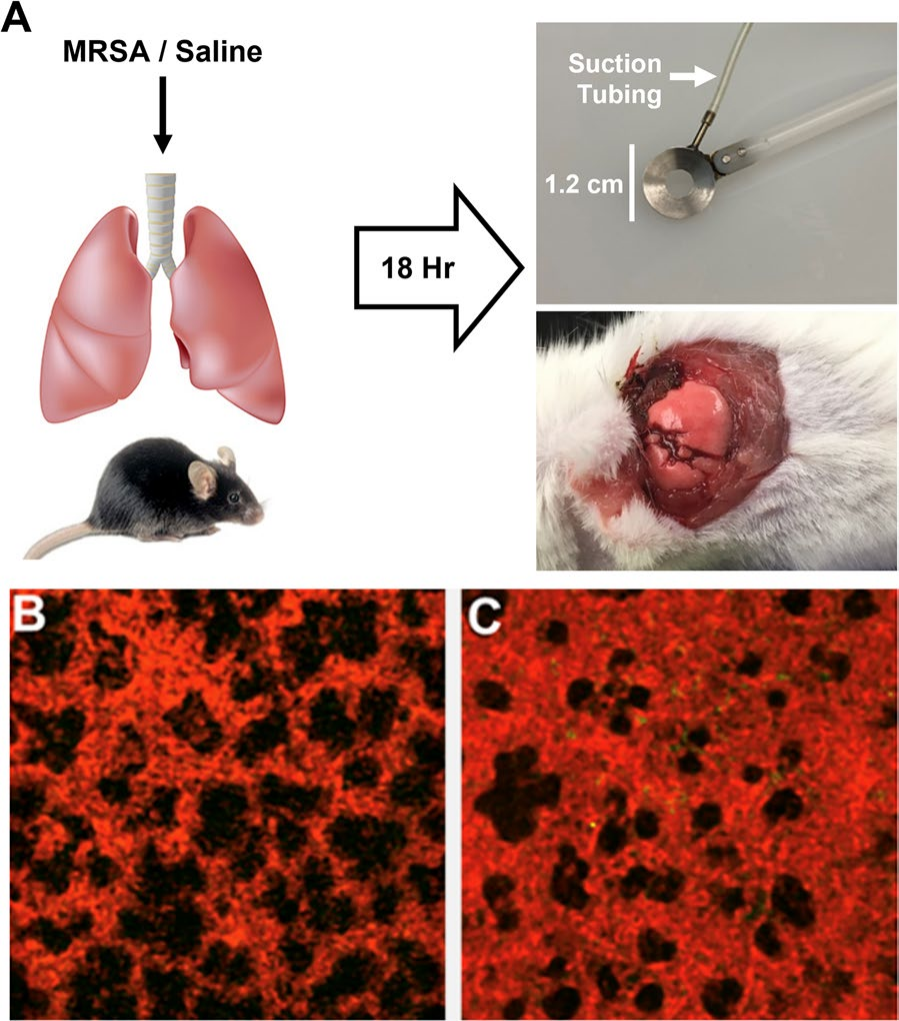
Experimental design and raw data acquisition. **A** Schematic representation of a murine model of inflammatory lung injury. Mice are administered intratracheal heat-killed methicillin resistant staph aureus (MRSA) or saline control. After 18 h animals are anesthetized, intubated and initiated on mechanical ventilation. A left lateral thoracotomy is performed to expose the lung surface which is overlayed with a custom made “lung window” (top right) equipped with suction and overlying coverslip. Two-photon intravital microscopy is performed to generate images in control or MRSA injured animals (**B–C**)

Intravital two-photon microscopy has proven to be an important technique to examine lung physiology in the murine system [8]. In these experiments animals are injected intravenously with fluorescent protein and antibodies to selectively label the vascular/interstitial space and infiltrating leukocytes respectively. Following intubation and initiation of positive pressure ventilation left lateral thoracotomy exposes the lung surface. Next, a custom-made 1.2 cm diameter “lung window” equipped with gentle suction and a glass coverslip is applied to the exposed lung tissue and held in place by a clamp stand. The lung surface is captured against the coverslip (Fig. 1A) as described previously [9]. In our lab, the lung is imaged using an Ultima In Vivo Microscopy System (Prairie Technologies) with a 60 × Nikon water immersion objective and high-speed resonant scanner. Time series images are obtained over several minutes and the animal is sacrificed at the conclusion of the experiment. Real-time acquisition of images from the intact, perfused and ventilated lung enables the investigator to identify changes to the vascular, interstitial and alveolar airspaces as well as characterize the presence and location of inflammatory cells [9, 10]. However, cyclical respiratory motion presents a considerable challenge to image analysis in the functional lung. These issues have been addressed through selective image acquisition and extraction during specific phases of the respiratory cycle in order to “stabilize” the image and improve the visualization of pulmonary vasculature and leukocyte infiltration [11] at the cost of losing information regarding alveolar mechanical processes. Others have employed intravital microscopy to measure alveolar dynamics in lung injury [12–14] but these techniques require tedious identification and measurement of individual alveoli which may give an incomplete picture of the integrative mechanical, vascular and inflammatory processes occurring simultaneously in the intact lung. In addition, these techniques can require the processing of thousands of image files to generate a useful video and prove to be labor intensive. Furthermore, identification and quantification of specific image features using these techniques is inherently subjective.

Several existing tools can handle either the image processing component [15–17], or basic forms of the time-dependent analysis component [18–22], sometimes at the level of a single image at a time, but do not support time-dependent analysis of these data. In terms of related, integrated systems for image processing and analysis, LIFEx [23] is an interactive visualization tool that can handle 3D MR and CT image data, and Imaris (Oxford Instruments) processes and reconstructs 3D cell data for analysis. However, these systems do not aim to support temporal data analysis. Other specific-purpose tools have been proposed to handle various time-dependent analysis problems in biomedicine [24–26], but are not extensible to our data and problem.

Driven by these limitations in the state-of-the-art, we developed a web browser-based interactive tool, Alveolus Analysis, for lung intravital microscopy image data preprocessing, analysis, and visualization. Alveolus Analysis provides a compact yet complete presentation of the physiologic information of interest. Features describing important respiratory and cellular details are extracted automatically before being arranged in a manner conducive to both intra-cycle analysis and inter-cycle comparison. This multi-level analysis allows for a comprehensive understanding of the time series data, beyond what is possible using currently available techniques.

## Implementation

Alveolus Analysis is a web browser-based application for the analysis and visualization of intravital lung microscopy imaging data. The tool’s design was inspired primarily by an activity-centered paradigm [27] with technical details included based on interviews with an experienced lung research team. A functional specification was used to further outline the tool’s layout and usage. The final product was arrived at after several iterations of parallel prototyping, hallway usability testing, and evaluation, culminating in a case study.

**Fig. 2:**
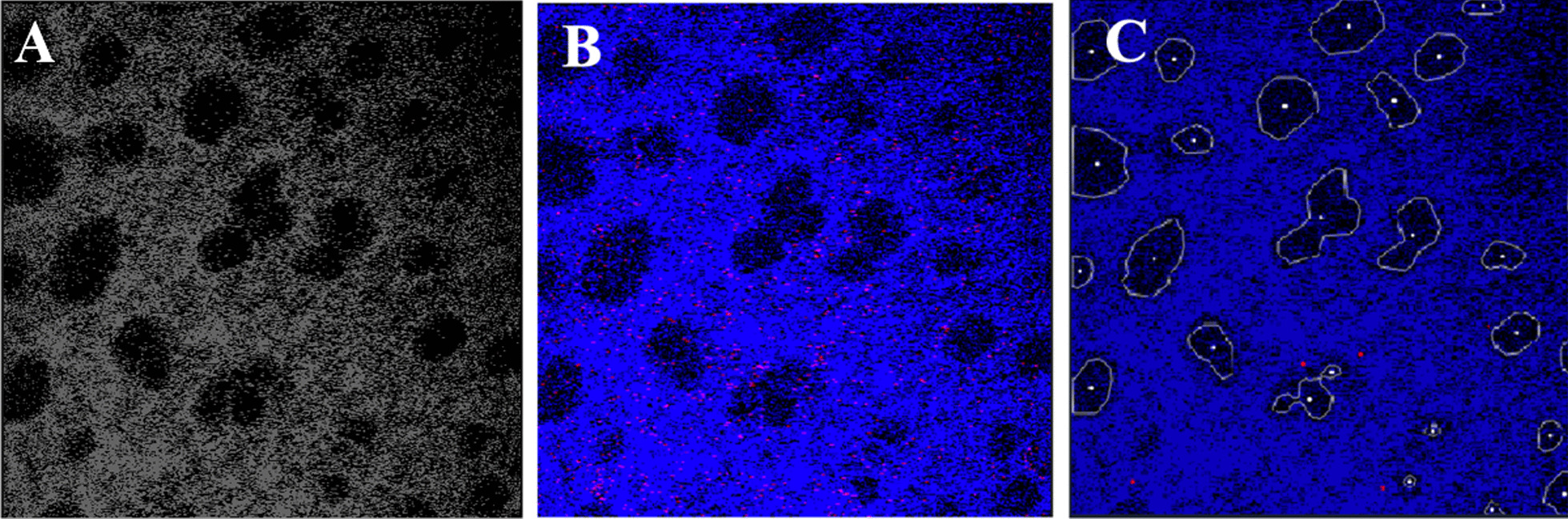
Image preprocessing. **A** Raw image of lung intravital microscopy data (black and white). **B** Processed intravital microscopy image shows the interstitial space (labeled with fluorescent dextran) highlighted in blue and neutrophils (stained with a fluorescent Ly-6 antibody) appear red. **C** Feature-overlayed image showing individual alveolar contours outlined in white and the position of neutrophils in red

There are two significant components to Alveolus Analysis’s architecture: (1) The pre-processing procedure, and (2) The web-hosted visualization. For pre-processing, code was developed to combine the separated-channel images of lung microscopy data into a single-color image (Fig. 2). The original format consisted of two separate images each containing a detected anatomic or physiologic feature, which we refer to as a “channel”: interstitial space and alveoli, or neutrophils. Combining these two images involved stacking these channels and assigning each one a color, drawing inspiration from how standard computer images are represented by three color channels “RGB”.

Furthermore, code was developed to extract various high-level features from the sequences of combined images. Of greatest importance is the feature extraction tool for alveoli, neutrophil, and interstitial areas. A series of denoising, blurring, and intensity filtering steps are used to eliminate as much noise from the images as possible. Specifically, non-local means denoising [28] removed noise across the entire image while classic denoising techniques of Gaussian blurring and intensity filtering [29] removed noise from the features. Denoising used a filter strength of 35, template size of 7, and search size of 21 for alveoli and filter strength of 45, template size of 7, and search size of 21 for neutrophils. For feature specific noise reduction, a blur kernel size of 11 and dilate kernel size of 4 were used. Further preprocessing parameter information along with recommendations and explanations can be found in the code repository (Additional file 1: Alveolus Analysis README). A classic edge detection algorithm is then used to provide contours of features within the image [30] (Fig. 2C). To avoid the detection of false positives, area restrictions were applied. Note that alveoli and neutrophils are processed and detected separately as their properties within the images are drastically different. In addition, a filtering technique was developed that could track alveoli and/or neutrophils over time. Stored areas and locations (according to contour centroid) are accessed at each frame of the video. A tracking requirement was used to determine if the detected feature was valid or not. In order to pass this requirement, the detected feature had to 1) exist in at least one of the neighboring frames in the video, and 2) match the area with some tolerance for growth/shrinkage (for alveoli) or with some fixed small tolerance (for neutrophils). These requirements prevented the appearance of falsely detected features and greatly improved alveoli and neutrophil detection accuracy. This usage of temporal dependencies present in the data marks a significant departure of Alveolus Analysis from other state-of-the-art static image processing tools.

Users first must run the preprocessing code on local computers where the image data is initially located. Then, upon completion of preprocessing, the newly generated images and features must be uploaded to the web application. Finally, the uploaded data, along with any other saved data, can be accessed within the web application itself. Steps within the application allow for users to choose which data to analyze in the data visualization.

The application was created using HTML, JavaScript, and D3.js and it is supported by a preprocessing backbone built using Python and OpenCV to ingest raw microscopy data, enhance and combine interstitial space and leukocyte imaging channels, and segment and extract feature-level information for alveoli and leukocytes. An example instance of the application is hosted at: https://github.com/uic-evl/AlveolusAnalysis.

## Results and discussion

**Fig. 3:**
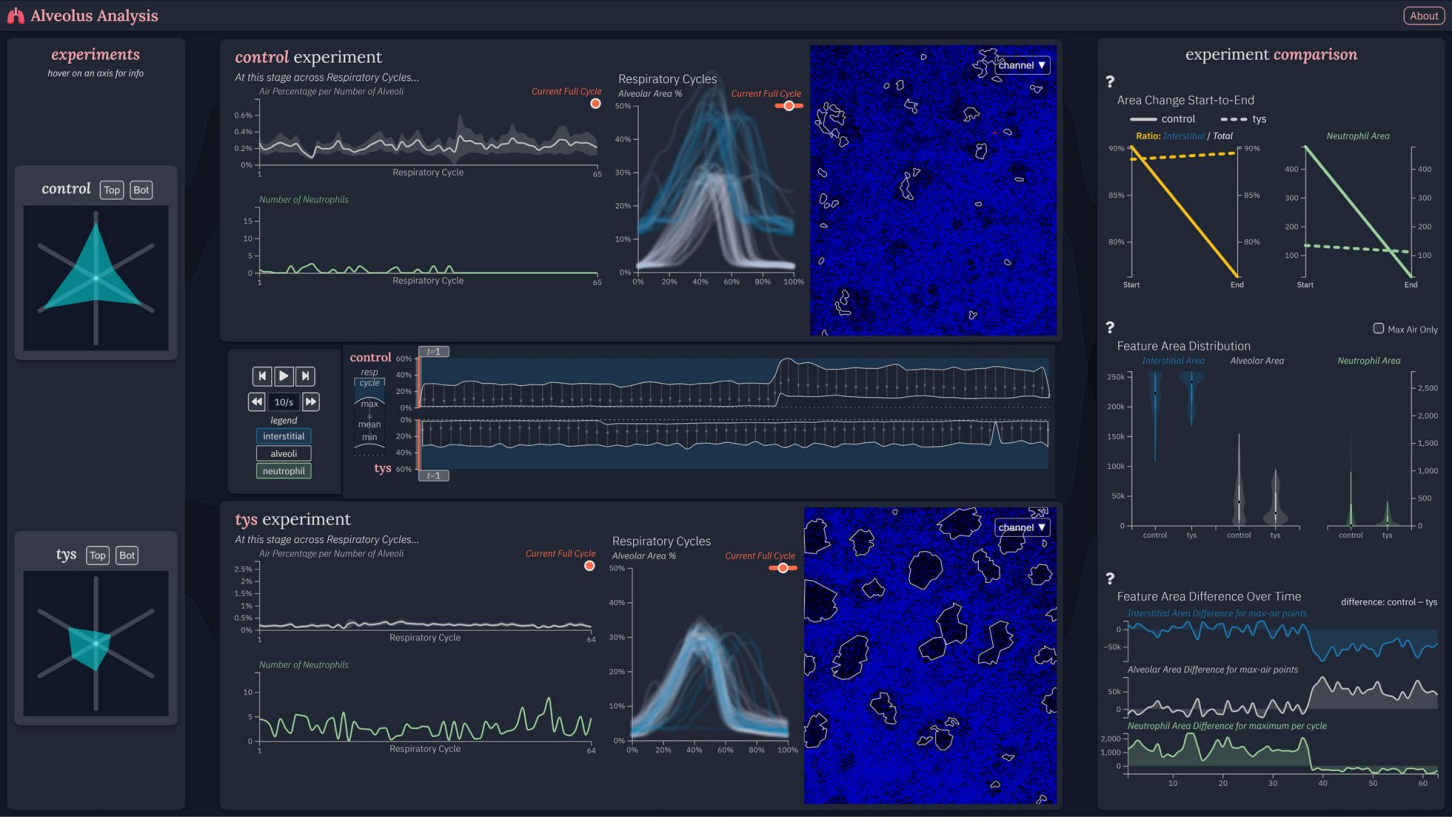
Screenshot of the web-based tool interface

The overall structure of the Alveolus Analysis interface (Fig. 3) attempts to fluidly connect all information needed by users to characterize lung edema and inflammation. Information is divided into three sections, each presenting a different level of detail and a corresponding level of interaction. This multi-level design, together with the connections between the components, smoothly connect values seen in one section to related reasoning in another [31, 32]. Comparison [33, 34] is a major functionality provided by the visualization and is intuitively integrated throughout the interface. Clearer separation is shown in the left and central components before distinct comparative plots and images are shown on the right, neatly summarizing the information in the analysis.

**Fig. 4:**
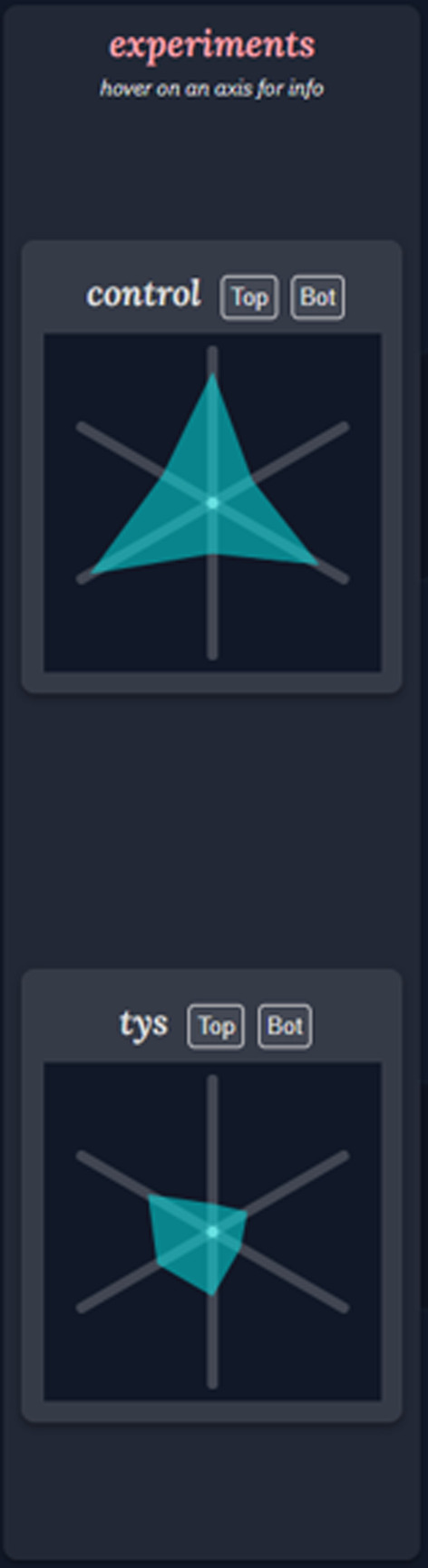
Navigation Bar. Experimental datasets are represented by descriptive statistics summarizing temporal alveolar or neutrophil behavior via a Kiviat diagram

On the left side of Alveolus Analysis interface, a user may select two experimental datasets to load simultaneously into the application (Fig. 4). A user's choice of experiment is informed by descriptive statistics which summarize temporal alveolar or neutrophil behavior, visualized in a modified Kiviat diagram [32, 35] to allow for shape-based inspection and comparison. This section is designed to be the starting point for the tool and allows the user to choose which set of images to analyze or compare. Critical summarizing information is shown to assist this choice. Faded graphics help guide users from this section to the middle section where experiment-specific information is presented.

**Fig. 5:**
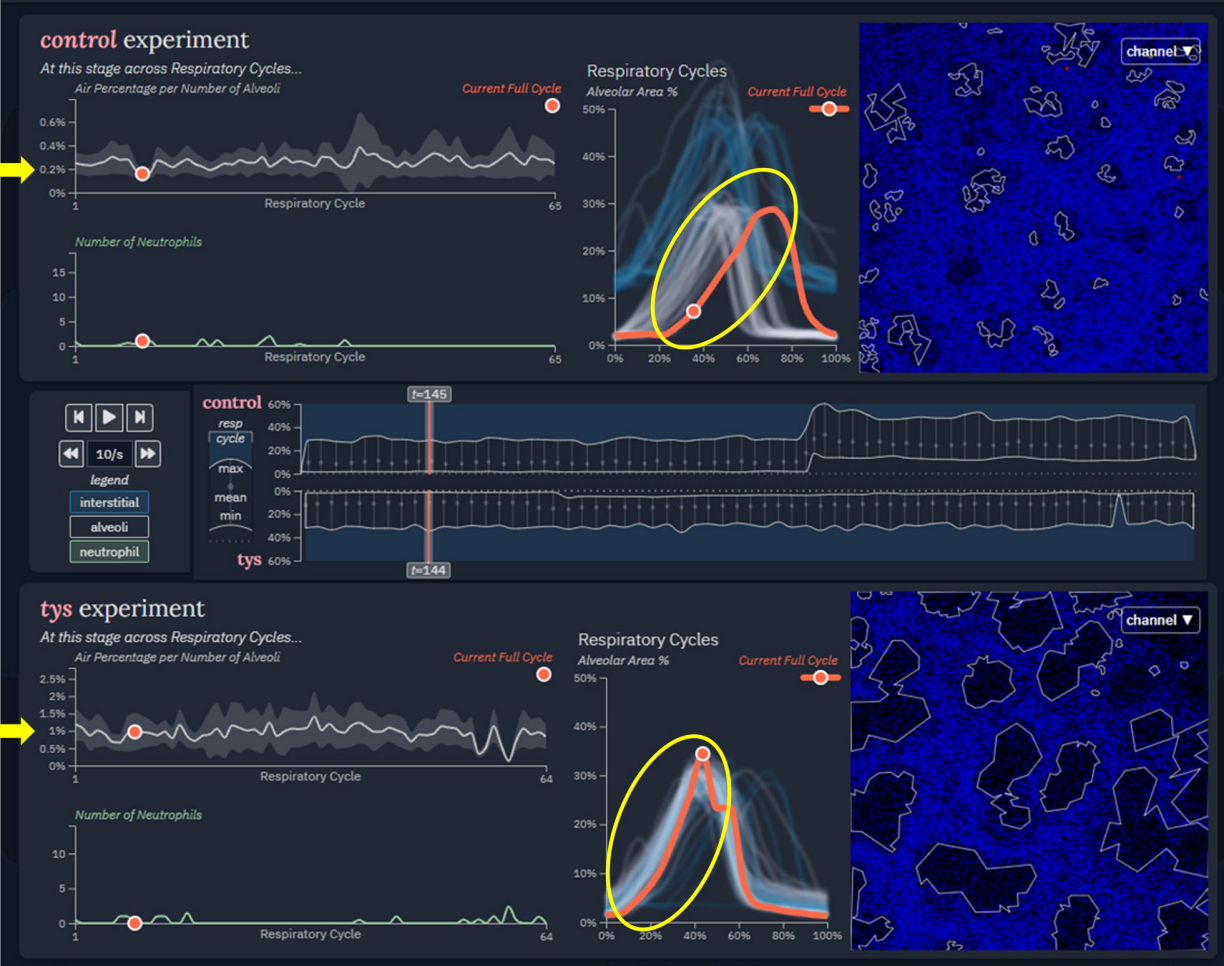
Central experimental data display. Two selected image series are depicted separated by mirrored timelines. Controls allow for offset or synchronized playback. Extracted information within each experiment is displayed across different temporal subdivisions. Differences in the amount of airspace area per alveolar number between control and Tys treated animals is highlighted (yellow arrows). The alveolar area percentage increases during inspiration (yellow ovals). The slope of this change may reflect differences in lung compliance between control and Tys treated subjects

In the middle section, more detailed values and statistics are presented corresponding to the selected experiments (Fig. 5). In keeping with the theme of smoothly transitioning from more broad information to more specific, analysis-friendly data, the middle section is divided vertically according to experiment. The two selected image series are arranged vertically within the interface, separated by mirrored timelines [33, 36] which depict long-term temporal behavior in relevant variables. These timelines show the minimal and maximal alveolar area percentage for each respiratory cycle and allow for global navigation through time. Time controls support playback of the experiments in tandem while also allowing a user to manually offset the time of one or both experiments to synchronize the respiratory cycle between experiments.

**Fig. 6:**
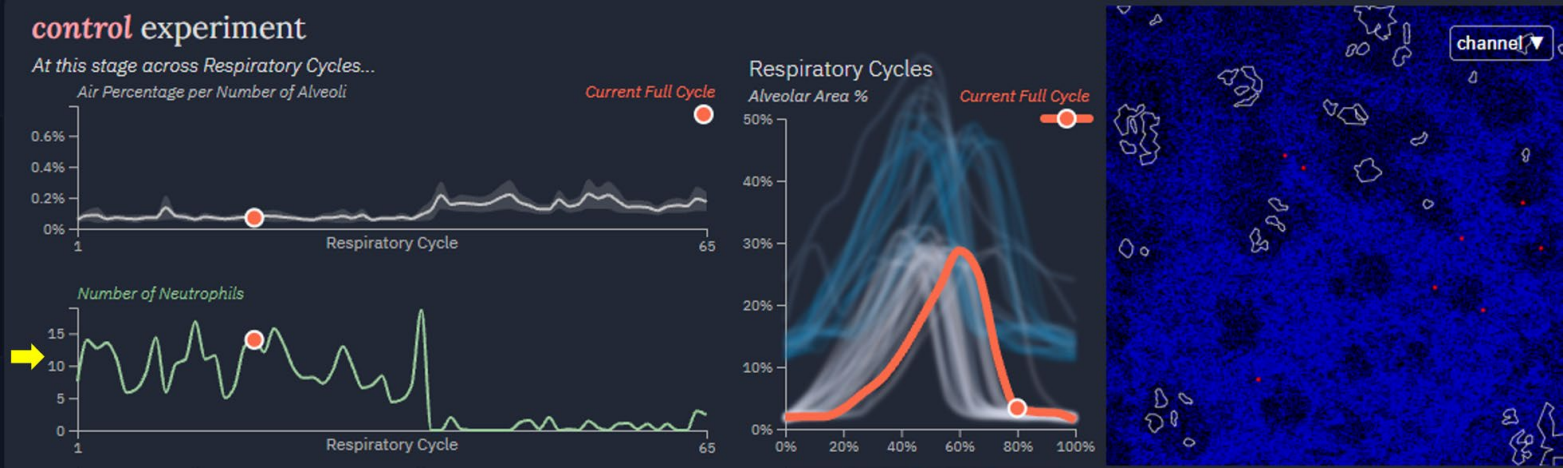
Individual time series data display. Alveoli, neutrophil, and respiratory cycle data are provided across different temporal subdivisions. The number of software identified neutrophils at similar points in each respiratory cycle is graphed across several cycles (yellow arrow)

For each image series, the application presents a user with three views (Fig. 6), from left-to-right: feature focused charts which highlight alveolar area percentages and neutrophil counts at the current respiratory cycle across the entire duration of uploaded data; a plot of overlaid respiratory cycles to highlight patterns and inconsistencies between a subjects tidal breaths; and the processed image at the current timestep overlaid with alveolar contours in white and the position of individual neutrophils in red. Again, details are presented in a manner that flows from more to less abstract. This assists users in digesting the complexity of the data [31] while maintaining the quality of the presented information. Important behaviors in the feature focused charts on the left naturally allow for investigation of related respiratory cycles in the middle before the lowest level of details are given on the right. Users may interact with a tracker in the left and the respiratory cycle graphics in the middle to quickly jump to other noteworthy areas of the data if necessary. If alternate image channels at a given timepoint are desired, that option is provided on the right, allowing for greater clarity either in the raw image data or with overlaid features.

**Fig. 7:**
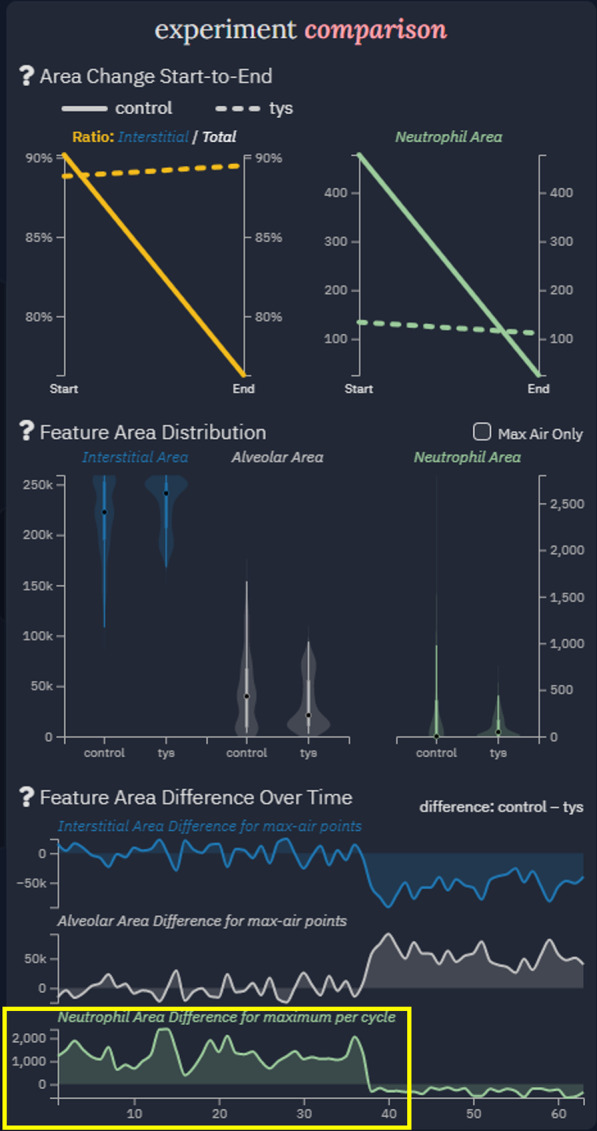
Summary comparison display. Extracted data is shown in a format conducive to analysis and comparison over the course of an entire series (top) as well as summarized variables between two experimental conditions (middle) and at specific time points between two individual time series (bottom). The difference in neutrophil area between the control and Tys treated animals is highlighted over the first 40 respiratory cycles (yellow box)

The middle section of the visualization is the primary focus of the tool [37]. It is connected via faded graphics to the right section which provides overarching information on the selected time series and additional comparisons summarized within singular plots. On the right-hand side of the interface, three charts further support explicit comparison of the selected image series based on temporal feature behavior (Fig. 7). The slope chart highlights the change in alveolar area ratio and neutrophil area from start to end of the time series, the violin plots visualize the distribution of feature area values between image series, and the difference line chart computes and presents the absolute difference in feature area in each aligned respiratory cycle between different image series. The emphasis on explicit comparisons allows users to quickly confirm or deny conclusions regarding the experiments. While not necessarily tied to any specific point in time, they give enough information to either quickly convey a discrepancy or verify a hypothesis in the analysis. Finally, users may access a description through a button at the top of the visualization. This feature is designed to help label important areas of the visualization and start users at the right locations for desired information. It also describes how to use the visualization’s interactive components, ensuring the tool is used to its fullest extent.

A case study was performed on the utility of the visualization. Two researchers with several years of experience in the field (PB and YMT) provided data and interacted with the tool. The target task was to compare image series data from two mice treated with intratracheal heat-killed staph aureus (hkMRSA) to induce lung inflammation [38]. The details of the experimental procedure are provided in Fig. 1. Two image series were preprocessed: one from a mouse pretreated with an experimental compound, FTY720 (s)-phosphonate (Tys), which was previously shown to ameliorate vascular leak during inflammatory lung injury [39–41] and another from a control (saline pre-treatment) animal. The datasets were loaded into the tool along with their extracted features.

The control and Tys data were automatically selected and displayed by the tool. Analysis thus began at the center of the tool, noting the timeline and embedded feature information across the entire time series. Attention then shifted to the respiratory cycle plots above and below the timeline, corresponding to each experimental condition. The team members noted that, although the visualization did not explicitly identify inspiratory and expiratory portions of the cycle, the tool was able to display them implicitly through detection and plotting of alveolar area. This was of particular use because in manual analysis this feature information would have been extremely difficult to extract from the video frames themselves. Furthermore, it was noted that the respiratory cycle plot essentially characterized the same information as typical volume over time plots obtained clinically during pulmonary function testing or viewed on a mechanical ventilator graphical interface. These graphs have the potential to identify changes in respiratory mechanics such as lung compliance. As an example, our colleagues noticed that the inspiratory slope of the control animal was flatter on average than that of the Tys-treated animal (Fig. 5, middle panel, yellow ovals). The initial conclusion was that the Tys-treated animal exhibited improved lung compliance compared to control, consistent with prior evidence of protection from lung injury and supporting the initial hypothesis.

While analyzing the respiratory cycle plots and moving through the time series from each condition, useful trends were identified in the cross-cycle plots to the immediate left. Functionality was confirmed by checking if the cross-cycle alveolar area and neutrophil area behaved inversely from one another (e.g. neutrophil area increases during expiration while alveolar area decreases). In addition, the first researcher noticed how an increased neutrophil area correlated with greater interstitial area throughout the control condition. This trend was less pronounced in the Tys condition. The decreased number of neutrophils in the interstitial space of the Tys-treated animal suggested less inflammation as compared to control. This again supported the initial hypothesis. The researchers finished their analysis of the tool’s central section by verifying their expectation of more alveolar air space (reduced interstitial area) in the Tys condition. This was supported in the air space area per number of alveoli plot when assessed over several respiratory cycles with a Tys alveolar airspace percentage of ~ 1% and a control alveolar area percentage of ~ 0.2% (Fig. 5, left panel yellow arrows). One colleague also pointed out that these plots would be useful in analysis of longer experiments to measure changes over time, expanding the tool’s potential application to additional studies.

It is worth noting that during the case study different feature overlay options on the processed video images were used to visualize the detected alveoli and neutrophils. Expert 2 remarked that neutrophils seemed to fade in and out of the images but the software identified neutrophil features seemed to be more realistic in terms of number and area. This was confirmed as part of the feature filtering process used by the tool and indeed reflected more realistic values (Fig. 6, yellow arrow). The research team then took note of the summarizing information displayed on the right side of the tool. It was immediately stated that having access to such experiment summarizing and cross-experiment information was very helpful in quickly identifying experiment quality and performing individual experiment and/or comparative analysis. Specifically, a colleague identified that there was a greater presence of neutrophils, as measured by a difference in area, in the control as compared to the Tys treated animal (Fig. 7, yellow box). They stated that this parameter could be used to quantify inflammatory injury and the treatment effect of experimental compounds. More broadly, both researchers noted extreme shifts in plot trends, for example the sudden drop in the control condition line in the area change plots. Upon referencing the timeline and respiratory cycle plot, they noticed a sudden decrease in image quality during the control experiment. This introduced artifacts into the video and thus heavily degraded the quality of the extracted information. Conversely, the Tys condition lines in the area change plots stayed relatively steady, following expectations.

Overall, our colleagues were able to conclude that, based on this experiment, Tys treatment may be protective in MRSA-induced lung inflammation. A consistent lack of neutrophils and low neutrophil area in the Tys condition images and feature plots supported this conclusion. The difference in inspiratory slope between the control and Tys respiratory cycles (Fig. 5, middle panel yellow circles) implied altered lung mechanics with lower lung compliance in control animals, providing further support of the initial hypothesis.

Feedback from our experienced lung researchers centered on an appreciation for the quantification of the video information. Previous analysis was primarily done by individual observers and was thus susceptible to qualitative bias or human error. Through computational preprocessing, feature extraction, and visualization, useful information is more directly and accurately presented in a “helpful” format. Perhaps one of the most central benefits of the tool was the fact that it slowed down the videos and presented them in digestible image formats. Combined with an intuitive frame-stepping and playback system, analysis was easier to perform. Compared to other tools, this one was more robust and provided more information. The researchers complimented the adaptability of the tool, postulating that it could be used for visualization and analysis in other biological systems imaged by intravital microscopy such as differences in perfusion and inflammatory cell infiltration in the heart or brain following ischemic injury models. Overall, they found the visualization extremely helpful in performing lung intravital image analysis and identifying new insights from the raw data.

Our results show that Alveolus Analysis is a powerful visualization tool that allows translational researchers to quickly analyze complex visual and temporal data. It allows investigators to take video data, in this case lung intravital microscopy images, and visualize them in a digestible but informative format. As shown in the case study, the layout and visual components of the tool provide significant benefits to investigator analysis, by facilitating smooth transitions between different levels of information and reveals new insights through detailed plots and interactivity. The research team noted that the tool was helpful to not only lung researchers but could have broad application in many areas of translational biologic research requiring imaging or video analysis.

Overall, the presentation of biological feature information in the data visualization provides important insight at several levels of abstraction useful to domain experts. With video data and extracted information presented at the individual image level, respiratory cycle level, and total experiment level, accurate analysis is robustly achieved. In addition, the layout, verified in a case study, ensured that the data visualization was intuitive and usable by researchers from the target field. This stands as a statement towards the generalizability of the tool. Furthermore, the tool is capable of processing and serving large amounts of video data as demanded in an accessible, user-friendly, and informative manner, representing a significant improvement over previous static image processing tools. Most importantly, the data visualization achieves the intended goal of taking the raw experimental video data and presenting it in an easily understandable and uniquely informative way. The visual data components of images and temporal features were effectively extracted from the base data and displayed in ways new to the field. The use of several levels of representation described a new, informative means of describing lung intravital microscopy video data. Furthermore, the data visualization was able to display such information at all levels in an interactive and connected way, easily allowing for comparisons and intuitive analysis.

We note the existing state-of-the-art feature extraction process is manual and prone to qualitative bias and human error. Our system automates the existing manual feature extraction process using evaluated and published state-of-the-art image processing techniques. Compared to the previous standard procedure of manual feature extraction and disjointed video analysis, Alveolus Analysis presents a clear benefit as an automation and integrated visualization tool.

Despite Alveolus Analysis advantages we acknowledge several limitations. Currently the tool handles single batches of video data. Scalability to multiple videos at the same time is limited due to the computational complexity of image processing techniques and the large number of images present in typical raw image data from a single experiment. Alveolus Analysis also has limits in its data pre-processing. For example, it does not aim to handle sudden shifts in video quality or the presence of artifacts in the images. In general, Alveolus Analysis assumes the input data is reasonably clean. For quality image processing and feature extraction, we provide further recommendations to the user regarding tuning the pre-processing hyperparameters. Information about what each hyperparameter does, as well as suggested values/ranges, are given in a simple interface to help simplify the process as much as possible. Currently, completely automating the feature extraction would present a considerable challenge.

With respect to assumption, Alveolus Analysis expects data to be presented as a series of individual images organized in temporal order (as is standard for video data) but split into two grayscale/single color channels, each encoding a feature of interest (e.g. interstitial space/alveoli and neutrophils). For details see Additional file 1: Alveolus Analysis README. This is necessary for the preprocessing and feature extraction procedure. Otherwise, Alveolus Analysis is robust to variations in modern web browsers (Chrome and Firefox) and display hardware (smaller or larger displays). A web demonstration is hosted at the code repository website. Of note, application of the tool to new datasets would require users to download the software, add the new data to the downloaded data directories, and install and host a local server as instructed in the Additional file 1: Alveolus Analysis README. Alveolus Analysis can then be accessed by anyone with the associated link. Optionally, the tool with new data may be uploaded as a new online code repository and accessed by anyone with access to the repository. For details, please refer to the code repository (Additional file 1: Alveolus Analysis README).

## Conclusion

In conclusion, Alveolus Analysis is a web browser-based interactive tool for lung intravital microscopy image data preprocessing, analysis and visualization, providing a comprehensive presentation of the biological information of interest. Our software marks a significant improvement over the state of the art tools in its ability to automate processing and analysis of lung intravital microscopy images at a level not previously possible. While the image processing, video data visualization, and interaction techniques used are relatively orthodox, they have not been applied in this specific problem and at the detail demanded, particularly with respect to temporal features. In addition, the biologic application required a level of accuracy and feature extraction that, to our knowledge, has not yet been addressed by image and temporal visualization. Finally, Alveolus Analysis has great potential for use in a variety of related biologic and physiologic fields. As noted by the researchers in the case study, analysis of video data is common in many different areas of translational research, and it is expected that the tool could be used to great effect in assisting the image analysis of other organ systems.

## Supplementary Information

**Additional file 1. **Alveolus Analysis README. User guide and code repository for the web-based application.

## Data Availability

All data generated or analyzed during this study are included in this published article and its supplementary information files.

## Abbreviations

ARDS: Acute respiratory distress syndrome
hkMRSA: Heat-killed methicillin resistant staph aureus
Tys: FTY720 (s)-phosphonate
