# Supplementary material for: Alveolus analysis: a web browser-based tool to analyze lung intravital microscopy
Source: BMC Pulm Med. 2022 Dec 17;22:480. doi: 10.1186/s12890-022-02274-7 (PMC9759058; doi:10.1186/s12890-022-02274-7)
Supplement: Supplementary file 1 — Additional file 1. Alveolus Analysis README. User guide and code repository for the web-based application. [file 12890_2022_2274_MOESM1_ESM.pdf]

# Alveolus Analysis - Web-based Application User Guide

This README contains a user guide for the Alveolus Analysis web-based application. It also includes instructions for installing the software, processing your own image data set, and running the tool locally.

We provide a Web Demonstration of the system which includes data for two pre-processed experiments. This demonstration may be found at the link below:

***Link to Web Demonstration of System***

---

## Table of Contents

**User Guide**

- Chart Explanations

**Setup Instructions**

- Clone The Repository
- Starting the Web-based Application
- Data Preprocessing

**Libraries and Tools Used**

---

## Application User Guide

This section will give you an overview of the visualizations included in Alveolus Analysis.

### Charts and Their Explanations

#### Experiment Selection and Navigation

| Name | Example | Explanation |
| --- | --- | --- |
| Experiment Summary | 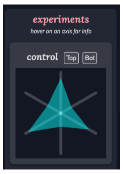  | A Kiviat diagram with summary statistics about each experiment to support visual comparison |
| Timeline | *wide image below* | The Timeline visualizes the change in alveolar/interstitial area over the course of each experiment. The blue area is the interstitial space, while the empty area is the alveoli. The stem represents the peak of each respiratory cycle and encodes the minimum, maximum, and mean observed alveolar area. You can use the play/pause controls to play the experiment like a video, or click/drag the timeline |

**Experiment Timeline**

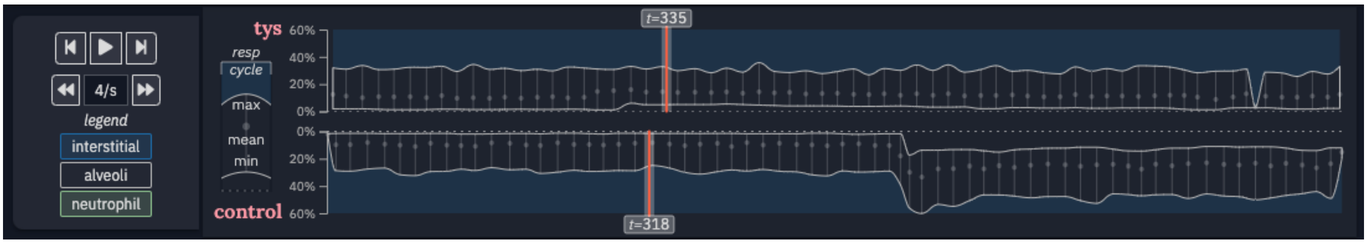


### Single Experiment Charts

| Name | Example | Explanation |
|---|---|---|
| Cycle Stage Over Time | 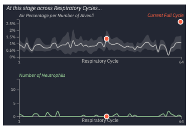 | The "cycle stage over time" chart visualizes the change in alveolar area and neutrophil count over the course of the experiment at a specific time point in a cycle. For example, if you have are viewing the point of peak inhalation of a respiratory cycle, this chart will plot these values for the peak of every cycle |
| Respiratory Cycle | 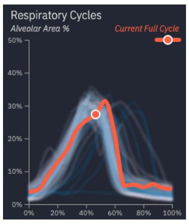 | The Respiratory Cycle chart overlays all respiratory cycles over the experiment, highlighting the current full cycle. Cycle curve colors fade from white to blue over the course of the experiment. The dot may be dragged horizontally to seek within a single cycle, and clicking on another cycle's curve will jump to view that time point |
| Image Preview | 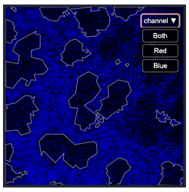 | The Image Preview shows the pre-processed, combined imaging data. White overlaid contours show the shape of extracted alveoli, and red dots show neutrophil features |

### Experiment Pairwise Comparison Charts

| Name | Example | Explanation |
|---|---|---|
| Area Change Start-to-End | 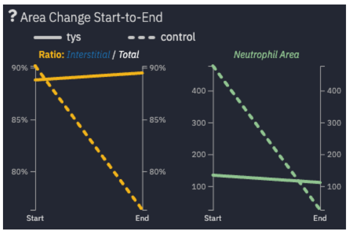 | The change in Interstitial % and Neutrophil area from the Start to End of the selected experiments |
| Feature Area Distribution | 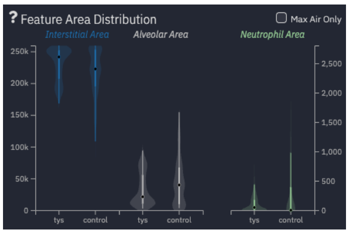 | The distribution of area by feature type across the selected experiments. Select the checkbox in the upper right to only show values at max inflation |

| Name | Example | Explanation |
| --- | --- | --- |
| Feature Area Difference Over Time | 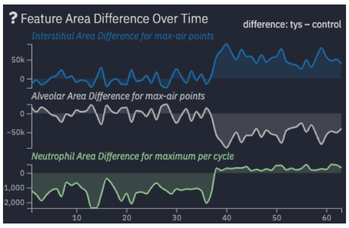 | The absolute difference in area by feature type between the two selected experiments |

## Setting up locally for your own use

This section will describe the steps to download the code of the project to your machine, pre-process your own data, and load it into the application

### Cloning the repository

You can download the code for this project by cloning the repository to your machine:

```
git clone https://github.com/uic-evl/AlveolusAnalysis.git
```

### Starting the Application

An easy way to use the application on your local machine is to use the Python `SimpleHTTPServer` as follows, then open your web browser (Chrome ideally) to http://localhost:8080

```
# inside the root directory of the repository
python -m SimpleHTTPServer 8080
```

### Data Preprocessing

#### Install Dependencies

```
pip install -r requirements.txt
```

#### Run the Image Processing

```
# parameter shorthand
python preprocess.py -m -p ./path/to/images/ -h 35 -t 7 -s 21 -g 11 -r 5 -a 15 -e 45 -w 7 -n 21 -d 4 -o 1 -l 10 -z 20
```

or

```
# parameter abbreviations
python preprocess.py --manual_mode --img_path ./path/to/images/ \
  --h_alv 35 \
  --tws_alv 7 \
  --sws_alv 21 \
  --gbks 11 \
  --thresh 5 \
  --min_area 15 \
  --h_neut 45 \
  --tws_neut 7 \
  --sws_neut 21 \
  --dks 4 \
  --ff_window 1 \
  --ff_loc_sense 10 \
  --ff_size_sens 20
```

#### Assumptions Made by Preprocessing Tool

The preprocessing tool makes several assumptions regarding the format of the stored data and where the outputs will be saved. Please ensure that the necessary steps are completed before running the above command(s). The assumptions are described below.

- Images are divided into two channels: ch2 and ch4, stored in two separate directories. These directories must be named exactly "ch2" and "ch4".
- Images are saved in the ".ome.tif" format and the archive is named following this general format: `<text>_Ch<2,4>_000001.ome.tif`
- A directory named "combo" exists in the provided image directory.

#### Input Image Data Examples

Below is an example of the input data for each `ch2` anc `ch4` image type.

> Note: the `ch4` image is very dark and has compression artifacts as shown below in .PNG format.

| `ch2` **image (Alveoli)** | `ch4` **image (Neutrophil)** |
| --- | --- |

| ch2 image (Alveoli) | ch4 image (Neutrophil) |
| --- | --- |
| 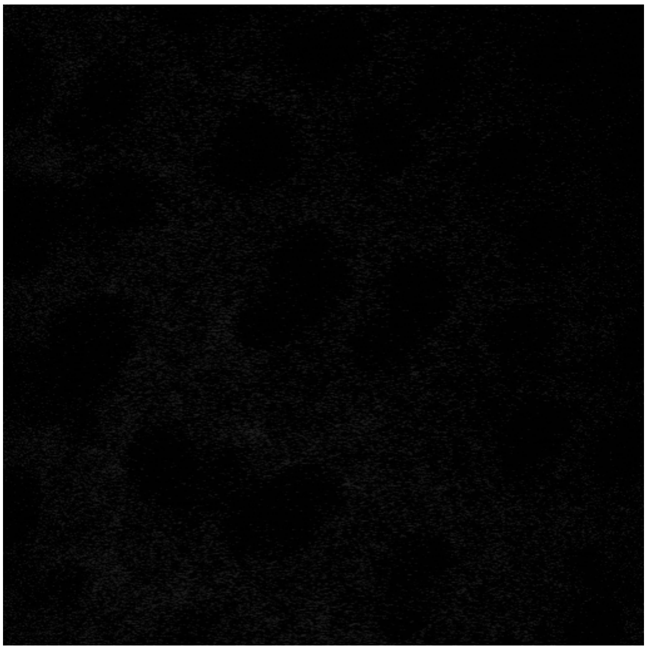 | 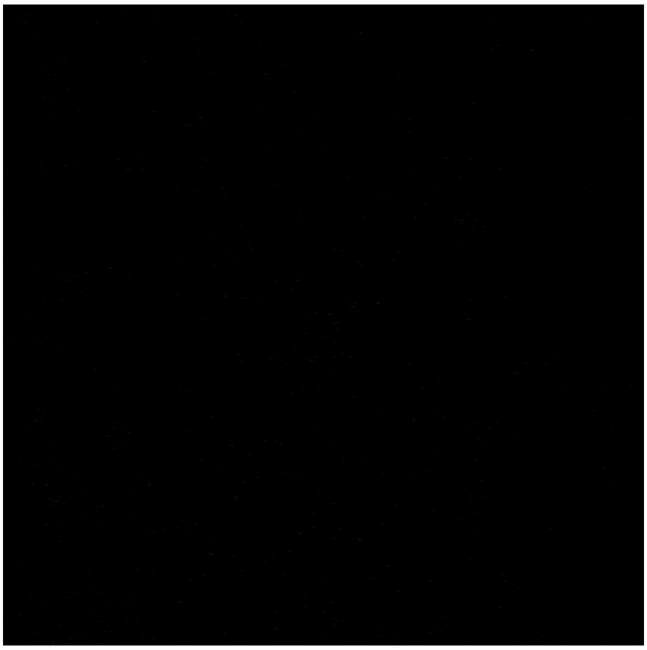 |

### Tuning Processing Hyperparameters

| Name | Abbreviation. | Shorthand | Feature Processing Stage (extraction or filtering) | Recommended Initial Value | Description |
| --- | --- | --- | --- | --- | --- |
| Denoising Filter Strength (Alveoli) | `--h_alv` | `-h` | extraction | 35 | removes noise from images but can also remove image details if set too high |
| Template Size (Alveoli) | `--tws_alv` | `-t` | extraction | 7 | must be odd number; area to calculate denoising operation, so a smaller value will focus on eliminating fine noise |

| Name | Abbreviation. | Shorthand | Feature Processing Stage (extraction or filtering) | Recommended Initial Value | Description |
|---|---|---|---|---|---|
| Search Size (Alveoli) | `--sws_alv` | `-s` | extraction | 21 | must be odd number; area to calculate averaging operation, so a smaller value will only use very close regions of image to fill in noise |
| Blur Kernel Size | `--gbks` | `-g` | extraction | 11 | must be odd number; area to blur image to further eliminate holes/edges caused by noise |
| Threshold | `--thresh` | `-r` | extraction | 5 | limit pixel intensity to keep in image, effectively eliminates noisy pixels leftover by the denoising and blurring operations |
| Min Area | `--min_area` | `-a` | extraction | 15 | minimum area a detected region must have in order to be maintained as a detected feature (**highly dependent on quality of videos**) |
| Denoising Filter Strength *(Neutrophil)* | `--h_neut` | `-e` | extraction | 45 | removes noise from images but can also remove image details if set too high |

| Name | Abbreviation. | Shorthand | Feature Processing Stage (extraction or filtering) | Recommended Initial Value | Description |
|---|---|---|---|---|---|
| Template Size *(Neutrophil)* | `--tws_neut` | `-w` | extraction | 7 | must be odd number; area to calculate denoising operation, so a smaller value will focus on eliminating fine noise |
| Search Size *(Neutrophil)* | `--sws_neut` | `-n` | extraction | 21 | must be odd number; area to calculate averaging operation, so a smaller value will only use very close regions of image to fill in noise |
| Dilate Kernel Size *(Neutrophil)* | `--dks` | `-d` | extraction | 4 | slightly expands detected neutrophil pixels to more accurately represent actual neutrophil areas |
| Feature Filter Window | `--ff_window` | `-o` | filtering | 1 | how many frames features must persist across (unidirectional) in both size and location |
| Filter Location Sensitivity | `--ff_loc_sens` | `-l` | filtering | 10 | how close feature centers should be in neighboring frames to be counted as the same, note that the unit here is pixels |

| Name | Abbreviation. | Shorthand | Feature Processing Stage (extraction or filtering) | Recommended Initial Value | Description |
| --- | --- | --- | --- | --- | --- |
| Filter Size Sensitivity | `--ff_size_sens` | `-z` | filtering | 20 | how close feature areas should be in neighboring frames to be counted as the same, note that the unit here is (approximately) square pixels |

## Libraries and Tools

Libraries Used for the implementation of the web-based application:

- d3.js (LICENSE): https://d3js.org/
- Popper (LICENSE): https://popper.js.org/
- Tippy.js (LICENSE): https://atomiks.github.io/tippyjs/
- FontAwesome Icons (LICENSE): https://fontawesome.com/
- favicon.io (LICENSE): https://favicon.io
